# Patient-Reported Knee Function Versus Star Excursion Balance Performance as Correlates of Psychological Return-to-Sport Readiness After Anterior Cruciate Ligament Reconstruction

**DOI:** 10.3390/bioengineering13091028

**Published:** 2026-09-03

**Authors:** Ryan T. Halvorson, Aidan Foley, Hayden Sampson, Cameron Nosrat, Drew A. Lansdown, Brian T. Feeley, Jeannie F. Bailey

**Affiliations:** Department of Orthopaedic Surgery, University of California San Francisco, San Francisco, CA 94143, USA

**Keywords:** anterior cruciate ligament, biomechanics, return to sport

## Abstract

**Background**: Psychological readiness, quantified by the Anterior Cruciate Ligament Return to Sport after Injury (ACL-RSI) scale, is one of the most consistent correlates of return-to-sport success following ACL reconstruction. Whether psychological readiness reflects an independent construct or is related to measured functional performance is not known. **Purpose**: To evaluate the associations between patient-perceived knee function and objective performance on a functional task, as quantified by traditional scoring, kinematics, and motion quality, and psychological readiness 9 to 12 months after ACL reconstruction. **Methods**: In this cross-sectional study, forty patients performed the Star Excursion Balance Test using a validated markerless motion capture system. Three biomechanical domains derived from that task (reach distance, lower-extremity kinematics, and a time-series motion quality index) were evaluated against psychological readiness, with patient-reported function (as measured by the Knee Injury and Osteoarthritis Outcomes Score [KOOS-12]) as a comparator. Associations were estimated using multivariable regression adjusted for age, sex, body mass index, and limb length. Relative contributions were evaluated with variance decomposition, and Bayes factors quantified the strength of evidence for or against each association. **Results**: Patient-perceived knee function was strongly associated with psychological readiness (standardized *β* = 0.58; 95% confidence interval, 0.29 to 0.87; BF_10_ = 249), whereas no biomechanical domain derived from the functional task reached statistical significance (all |*β*| ≤ 0.34; all |*r*| ≤ 0.3). In the combined model (total *R*^2^ = 0.463), variance decomposition attributed 89.3% of explained variance in psychological readiness to patient-perceived knee function, versus 6.9% for reach distances, 2.4% for motion quality, and 1.4% for lower extremity kinematics. Bayes factors favored the null for each biomechanical domain, but reached only anecdotal strength (BF_01_, 1.3–2.8). **Conclusion**: In this cross-section, psychological readiness after ACL reconstruction more closely aligned with patient-perceived knee function than biomechanical performance on the SEBT, indicating that perceived function and biomechanical performance on certain tasks may be divergent at the time of return-to-sport clearance. These findings support the routine inclusion of patient-reported outcome measures alongside functional testing in multi-domain return-to-sport assessment.

## 1. Introduction

There are over 200,000 anterior cruciate ligament (ACL) injuries per year in the United States [1]. Despite the implementation of more standardized rehabilitation protocols and return-to-sport testing criteria, up to 47% of athletes fail to return to sport at their preinjury level following anterior cruciate ligament reconstruction (ACLR) and 11% of athletes experience graft re-rupture [2,3,4,5,6]. As there are serious long-term consequences of ACL reinjury, including elevated risk for post-traumatic arthritis, worse patient-reported outcomes, and higher risk for additional reinjury, it is important to identify factors predictive of successful return to activity and graft re-rupture [7,8,9].

Many studies have failed to identify a clear link between physical recovery and successful return to sport, suggesting other factors may be more important than biomechanical or functional test performance [10,11]. Psychological readiness to return to sport has been proposed as a potentially modifiable and critical factor in guiding return to sport success, and is typically quantified using the ACL Return to Sport after Injury (ACL-RSI) scale [12]. Psychological readiness frequently demonstrates higher predictive value for return to sport success than other patient demographic, biomechanical, or functional variables [13,14]. Patients with lower psychological return to sport readiness are also at higher risk for graft rupture and contralateral ACL injury [15]. However, whether psychological readiness reflects a truly independent psychological factor or instead represents unmeasured confounding with patient-reported or functional performance measures has not been well established.

Existing literature has been mixed on the relationship between ACL-RSI and patient biomechanical and functional variables. In a cross-sectional analysis of 452 male athletes 9 months after ACLR, O’Connor found no relationship between psychological readiness and athlete strength or power measures, including isokinetic strength testing and jump testing [16]. At 12 months following ACLR, Cronström et al. identified a weak association between single-leg hop distance and ACL-RSI, but found no relationship between ACL-RSI and other functional test performance, including muscle strength and range of motion [17]. Given the weak and inconsistent relationships between ACL-RSI and these limited functional assessments, it is important to consider other possible explanatory factors including more advanced biomechanical assessments.

The kinematic deviation index (KDI) is a comprehensive motion assessment derived from three-dimensional posture modeling that quantifies patient motion quality during a functional assessment [18,19]. It has previously been shown to discriminate patients with lower extremity pathology, and correlates with patient-reported outcomes [18]. Evaluating patient motion quality and patient-reported symptoms could help disentangle whether psychological readiness is better explained by their own perception of their knee or how it performs in an objective biomechanical assessment.

We comprehensively phenotyped affected-limb function 9–12 months after ACLR using a validated markerless motion-capture system including functional test performance, lower extremity kinematics, and a time-series movement-quality index (KDI). We then tested each domain against psychological readiness, as measured by ACL-RSI, alongside patient-reported knee function (as quantified by the Knee Injury and Osteoarthritis and Outcomes Score [KOOS]) as a comparator. The purpose of this cross-sectional study was to evaluate the associations between patient-perceived knee function and objective performance on a functional task, as quantified by traditional scoring, kinematics, and motion quality, and psychological readiness 9 to 12 months after ACL reconstruction. We hypothesized that psychological readiness would be strongly correlated with patient-perceived function but not biomechanical performance on the functional task.

## 2. Materials and Methods

All study activities were approved by the University of California, San Francisco Institutional Review Board (23-40665). This exploratory, cross-sectional analysis included adult patients at their 9- and 12-month follow-up appointments following ACL reconstruction. All patients underwent ACL reconstruction with either an autograft or allograft, and no patients underwent primary repair. Patients were recruited during routine follow-up appointments. Patients were excluded who had a revision ACLR, contralateral ligamentous deficiency, multiligamentous knee injuries, or other medical conditions significantly impacting motion. All participants were managed with a criterion-based rehabilitation protocol, including range of motion, quadriceps activation, strengthening, running progression, and sport-specific phases.

### 2.1. Psychological Return to Sport Readiness

The primary outcome was psychological return to sport readiness, assessed using the ACL-RSI [12]. ACL-RSI has been established as an important predictor of both successful return to sport and reinjury risk following ACLR [13,14,15]. Patient-perceived knee function was assessed using KOOS-12, which is a self-administered assessment which incorporates knee-related pain, symptoms, impact on quality of life, activities of daily living, and sports and recreational function [20]. KOOS-12 has been previously validated in patients undergoing ACLR [21,22]. All patient-reported outcomes were collected at the routine follow-up appointments immediately after the biomechanical assessment by study personnel.

### 2.2. Biomechanical Assessment

**Figure 1:**
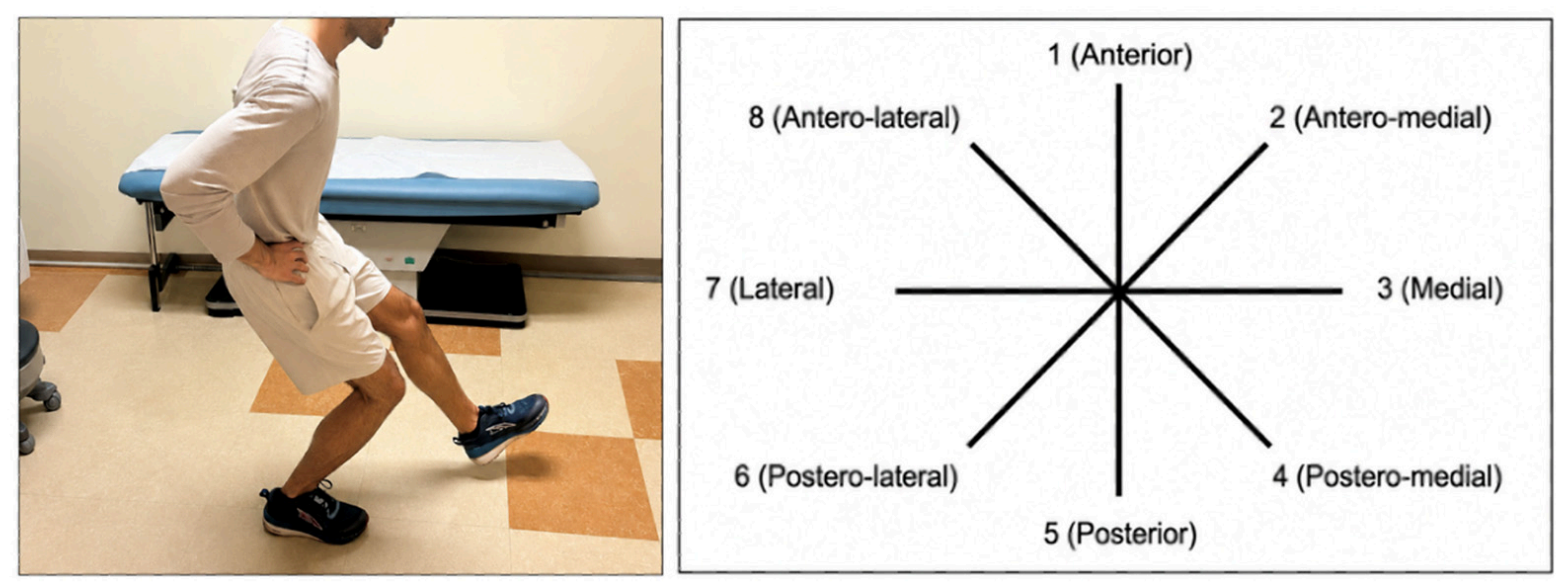
Star Excursion Balance Test (SEBT) Configuration. The top panel depicts an actor performing SEBT. The patient balances on the limb being evaluated and reaches maximally in each of the eight directions depicted on the bottom panel.

The SEBT is a lower extremity biomechanical assessment, in which patients balance on the leg of interest and reach maximally in eight directions around a star with the contralateral leg (Figure 1). This places high demand on patient balance and neuromuscular control, and requires both strength and range of motion to achieve high scores [23]. Performance on the SEBT has previously been proposed as a criterion for return to sport following ACLR [24,25]. Furthermore, the SEBT can be administered in a clinic without the need for specialized laboratory equipment, which supports its use as a point-of-care assessment. The SEBT is traditionally scored according to the maximum reach distance achieved in each of the eight directions. During SEBT, the patients were instructed to maintain their hands resting on their hips, and all trials were observed by a member of the study team. Patients who deviated from the guidelines were asked to repeat trials. All patients completed three repetitions of SEBT on each leg, including the operative and nonoperative extremity.

SEBT trials were recorded using a previously validated markerless three-dimensional motion capture system which uses a commercially available depth camera (Azure Kinect, Microsoft, Redmond, WA, USA) [18,19,26]. The camera emits infrared light and reconstructs a depth map from the phase signal of the returned infrared signal, ultimately returning three-dimensional coordinates for 32 body landmarks which have been validated against marker-based systems in functional tasks [27]. All joint position data were filtered according to previously validated, allometrically scaled models [26]. SEBT reach distances were calculated as the maximum distance between the stance ankle center and reach ankle center along each reach vector during each reach attempt. Hip, knee, and ankle segment angles were calculated in the coronal and sagittal planes at the points of maximal reach during each SEBT trial, as this represents maximal patient exertion.

Because reach distances are only an indirect proxy measure for the complex strength and neuromuscular control required to perform SEBT, a comprehensive motion quality index was also used to score patient performance during the assessment. For this purpose, the KDI was selected, as it is a comprehensive motion assessment derived from three-dimensional posture modeling that quantifies patient motion quality during SEBT [18,19]. Technically, it is an advanced statistical assessment of time-series 3D joint position data which quantifies postural deviation during a task from a theoretical ideal motion trajectory. It has previously been shown to discriminate patients with lower extremity pathology during SEBT, and correlates with patient-reported outcomes [18]. Similar metrics have also been used to evaluate patient performance in other patient cohorts and during similar tasks [28,29].

### 2.3. Statistical Approach

Biomechanical variables were categorized into three domains: functional test performance (SEBT reach distances), biomechanical performance (joint angles), and motion quality (KDI). Patient-perceived function, as quantified by KOOS-12, was used as a comparator. Given there were three reach attempts per limb per participant, the within-subject reliability for each biomechanical variable was assessed using a two-way, absolute agreement, random effects intraclass correlation coefficient (ICC 2,3), with the three trials as repeated measures.

The association between each biomechanical domain and ACL-RSI was first evaluated using a multivariable regression model adjusted for age, sex, BMI, and limb length. The primary biomechanical variables used in this analysis are from the operative limb unless otherwise specified. Because bilateral data were available, limb symmetry indices (operative limb as a ratio of nonoperative limb, expressed as a percentage) were additionally computed for reach distance, joint kinematics, and the motion quality index. Associations are reported as the effect size with 95% confidence intervals alongside the adjusted correlation coefficient, *R*^2^. All variables were standardized (mean 0, SD 1) so that coefficients represent the standard-deviation change in ACL-RSI per standard-deviation change in the covariate. Regression assumptions were evaluated for all models. Linearity and homoscedasticity were assessed using residual-versus-fitted plots and the Breusch–Pagan test, normality of residuals using quantile-quantile plots and the Shapiro–Wilk test, influential observations using Cook’s distance, and multicollinearity using variance inflation factors.

To evaluate relative factor importance, all variables were included in a single model and LMG decomposition was performed [30]. To evaluate for absence of association, Bayes factors were computed for each biomechanical domain. Bayes factors were interpreted using conventional thresholds, with BF_10_ values between 1 and 3 considered anecdotal evidence, 3 to 10 moderate, 10 to 30 strong, and greater than 30 very strong evidence in favor of an association [31].

Statistical significance was set at *p* < 0.05. A power analysis indicated that a sample of 40 patients provided 80% power to detect a correlation of |*r*| ≥ 0.43 (assuming two-tailed α = 0.05) [32]. The power to detect an individual coefficient after adjusting for covariates was comparable (partial r ≥ 0.42). The study was therefore powered to detect moderate-to-large associations, but smaller associations between biomechanical measures and ACL-RSI cannot be excluded. All analyses were performed using R version 4.4.1 (R Foundation for Statistical Computing, Vienna, Austria) [33].

## 3. Results

### 3.1. Cohort Demographics

Sixty patients were recruited sequentially at 9- or 12-month follow-up appointments and were eligible for inclusion. Twenty patients declined participation or were unable to remain for testing, resulting in 40 patients available for analysis. There were 18 (45%) males; the average age was 32.7 years (standard deviation [SD] 10.5), and the average body mass index (BMI) was 25.9 (SD 3.7, Table 1). Thirty-two patients (80%) received autograft ACL reconstructions, including 23 hamstring grafts, seven quadriceps grafts, and two bone–tendon–bone grafts. The mean graft diameter was 8.8 mm (SD 0.6). Six patients received a lateral extra-articular tenodesis. Twelve patients (30%) had lateral meniscus repairs and four patients (10%) had medial meniscus repairs. Twenty-three patients were recruited at the 9-month appointment (mean 37.1 weeks, range 30 to 44 weeks) and 17 patients were recruited at the 12-month appointment (mean 51.7 weeks, range 43–62 weeks). The mean interval from surgery to assessment was 43.3 weeks (SD 8.7; median 42; range 30 to 62). Individual visits occurred somewhat earlier or later than their nominal timepoint, as is usual in routine clinical follow-up.

### 3.2. Patient-Perceived Knee Function

**Figure 2:**
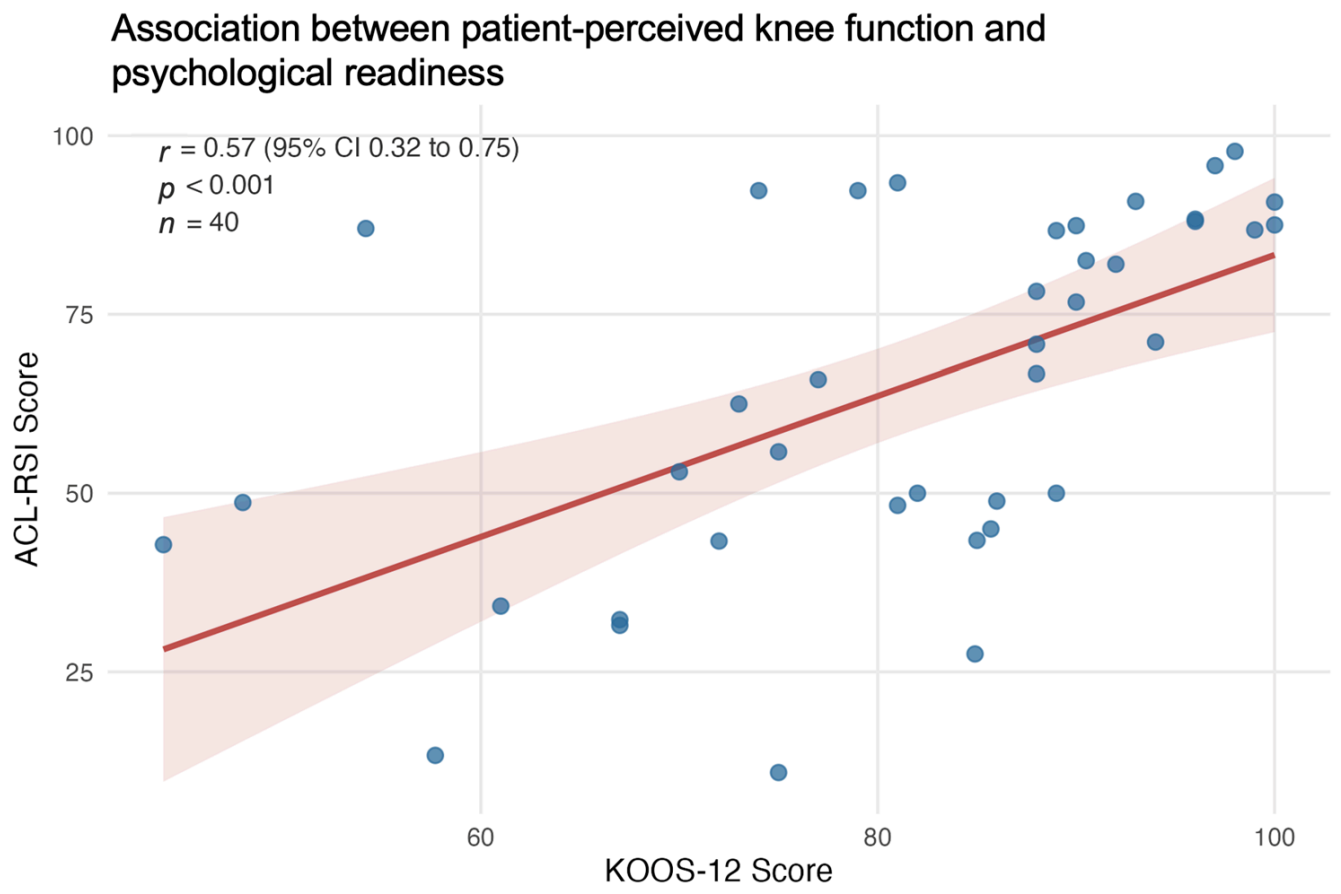
Association between patient-perceived knee function and psychological readiness. Correlation coefficient *r* = 0.57 (95% CI 0.32–0.75, *p* < 0.001). Adjusted *β* = 0.58 (0.29–0.87). Red line represents fitted least squares regression line of best fit and shaded area represents 95% confidence interval.

After adjusting for age, sex, leg length, and BMI in a multivariable linear regression model, patient-reported knee function (KOOS-12) was significantly associated with psychological readiness (standardized *β* = 0.58, 95% CI 0.29–0.87; *p* < 0.001; Figure 2). Age, sex, leg length, and BMI were not independently associated with psychological readiness (*p* > 0.05). The unadjusted correlation coefficient between KOOS-12 and ACL-RSI was 0.57 (95% CI 0.32–0.75).

### 3.3. Functional Test Performance

**Figure 3:**
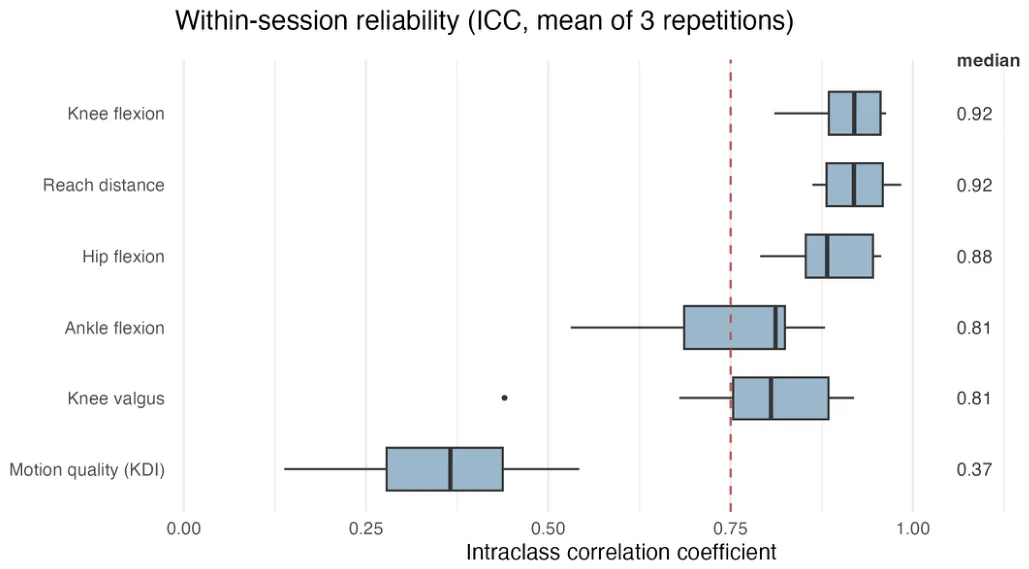
Within-session reliability for functional test performance. Individual point represents an outlier. Dashed line represents a reference at ICC = 0.75.

Within-session reliability (ICC for the mean of three repetitions) was good to excellent for reach distance (ICC range 0.86–0.98) and for sagittal-plane joint angles (knee flexion 0.81–0.96; hip flexion 0.79–0.96, Figure 3). Frontal-plane knee valgus and ankle flexion were more variable across reach directions (0.44–0.92 and 0.53–0.88, respectively). The motion-quality index (KDI) was also variable across reach directions (range 0.14–0.54), with higher reliability for directions 2 (0.54), 4 (0.42) and 8 (0.54).

**Figure 4:**
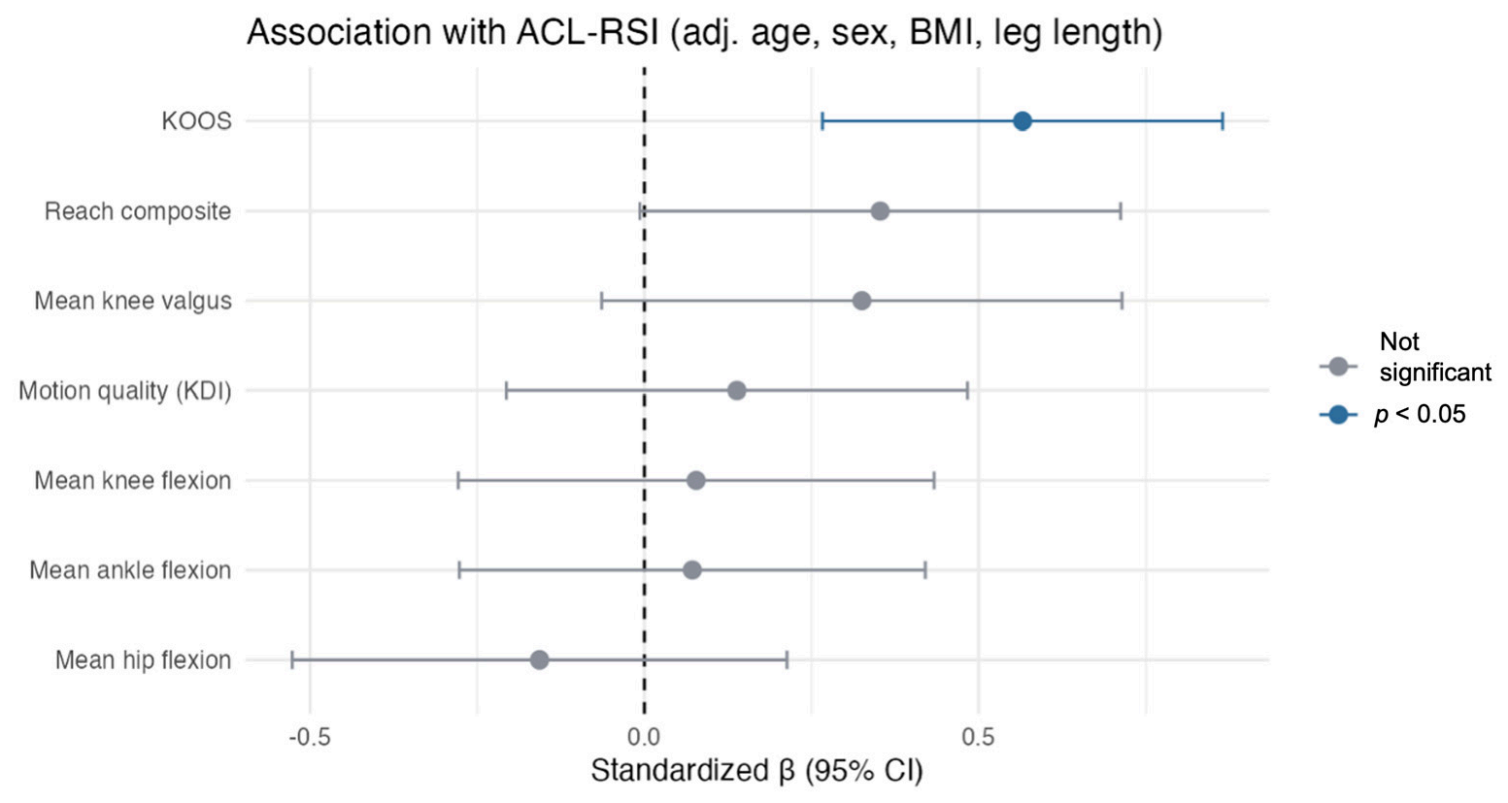
Forest plot demonstrating overall model fit for each biomechanical domain and psychological readiness. Dashed line represents *β* = 0 (no relationship).

After adjusting for age, sex, BMI, and leg length in separate multivariable linear regression models, no biomechanical or functional domain was significantly associated with ACL-RSI (Figure 4): SEBT reach composite (*β* = 0.34, 95% CI −0.02 to 0.70, *p* = 0.064), mean knee valgus (β = 0.32, 95% CI −0.07 to 0.70, *p* = 0.11), mean knee flexion (*β* = 0.08, 95% CI −0.27 to 0.44, *p* = 0.64), mean ankle flexion (β = 0.07, 95% CI −0.28 to 0.42, *p* = 0.68), mean hip flexion (*β* = −0.16, 95% CI −0.52 to 0.21, *p* = 0.40), or KDI (*β* = 0.11, 95% CI −0.23 to 0.46, *p* = 0.51). Across all eight SEBT reach directions, none of the individual reach distances, joint angles (knee, hip, and ankle flexion; knee valgus), or KDI values were significantly associated with ACL-RSI. All correlations between functional test performance and ACL-RSI were weak (|*r*| ≤ 0.3) with *p* > 0.05.

In addition to disease-side reach distances, the side-to-side difference between the surgical and healthy limbs was also calculated. Limb symmetry indices were computed for all biomechanical domains as exploratory. Reach distance symmetry was not significantly associated with psychological readiness (*r* = 0.30, *p* = 0.076), nor were symmetry indices for hip flexion, ankle flexion, or motion quality (all *p* > 0.13). Knee flexion symmetry showed a nominal association (*r* = 0.34, *p* = 0.046). In these models, age, sex, and BMI were not independently associated with psychological readiness (*p* > 0.05).

Regression diagnostics did not identify violations of model assumptions. Residuals were consistent with normality in all models (Shapiro–Wilk *p* = 0.08 to 0.66), there was no evidence of heteroscedasticity (Breusch–Pagan *p* = 0.45 to 0.55), and no observation approached an influential value (maximum Cook’s distance 0.49; no observation exceeded 1).

**Figure 5:**
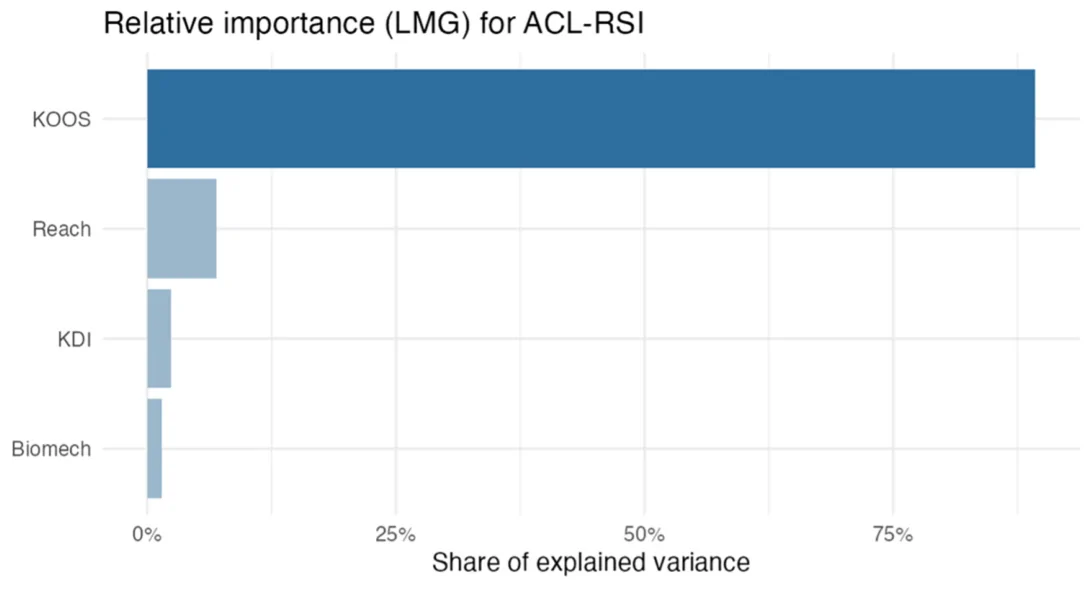
Relative importance decomposition of patient-perceived knee function and SEBT performance in a combined model. Dark blue represents patient-perceived function while light blue represents biomechanical domains including SEBT reach distance, motion quality (KDI), and lower extremity kinematics (Biomech).

In a combined, adjusted model containing all four domains, relative-importance (LMG) decomposition was performed (Supplementary Table S2). This attributed 89.3% of the explained variance in ACL-RSI (41.4% of total variance [LMG model *R*^2^ = 0.463]) to patient-reported knee function (KOOS-12), with reach distances (6.9%), motion quality (2.4%), and joint kinematics (1.4%) each contributing negligibly (Figure 5). These percentages describe the partitioning of the variance the model explains and should not be interpreted as partitioning of the total variance in psychological readiness. Variance inflation factors ranged from 1.2 to 6.2; the highest values were among the joint-angle composites, which are correlated with one another, whereas patient-perceived knee function was essentially independent of the biomechanical predictors (variance inflation factor 1.2). The individual model coefficients, confidence intervals, and variance inflation factors are reported in Supplementary Table S2. The correlation between all predictor variables is reported in Supplementary Table S3.

**Figure 6:**
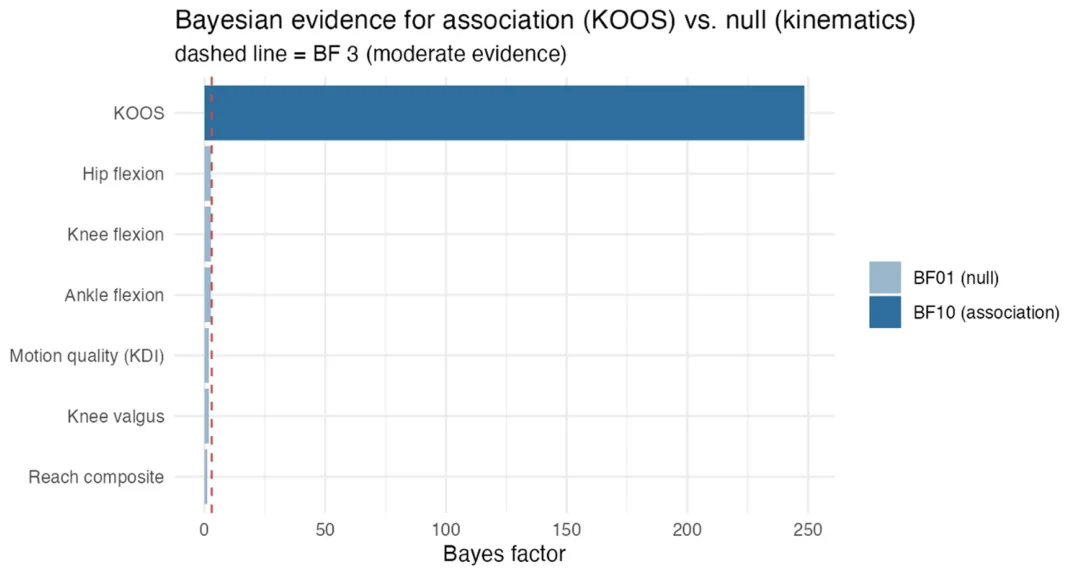
Bayes factors for each biomechanical domain.

Bayesian analyses reinforced the dissociation between biomechanical variables and functional test performance and psychological readiness (Figure 6). For patient-perceived knee function (KOOS-12), the Bayes factor indicated extreme evidence favoring an association with ACL-RSI (BF_10_ = 249, Figure 6). For every biomechanical and functional domain, Bayes factors favored the null over an association (BF_01_ 1.3–2.8) but did not reach the threshold for moderate evidence (BF_01_ > 3). These values therefore do not establish an absence of association.

## 4. Discussion

In this cross-sectional analysis of patients following ACLR, patient-perceived knee function was strongly associated with psychological readiness, while functional test performance, lower extremity kinematics, and motion quality were not. In the combined model, 89.3% of the explained variance in ACL-RSI was attributable to KOOS-12, versus 6.9% to SEBT reach distances, 2.4% to KDI, and 1.4% to lower extremity kinematics. These findings were consistent across multivariable regression, LMG decomposition, and Bayes factor analysis across all components of the functional task. Although Bayes factor analysis failed to reach a significance level for lack of association, this study did not identify an association between perceived and objective functional recovery at the usual time of return to sport decision making.

This study strengthens the argument for routine inclusion of patient-reported outcomes as part of return-to-sport clearance protocols following ACLR. Prior research has established psychological readiness as an important predictor of return to sport success and risk for reinjury, demonstrating higher predictive value than other patient demographic, biomechanical, or functional variables [13,14,17]. This study adds that psychological readiness itself, as quantified by ACL-RSI, is directly related to patient-perceived knee function. This finding brings into question the precise directional relationship and orthogonality of the KOOS-12 and ACL-RSI instruments. When interpreting the association between these patient-reported outcomes, including the LMG decomposition analysis, it is important to consider that both instruments were administered at the same time and have some degree of overlap in the content. KOOS-12 specifically assesses knee-related pain (4 items), function (4 questions), and impact on quality of life (4 questions) [20]. Although ACL-RSI directly addresses sport-related scenarios in most questions, there are two questions on the KOOS-12 survey that potentially overlap with psychological readiness, including “have you modified your lifestyle to avoid activities potentially damaging to your knee” and “how much are you troubled by lack of confidence in your knee”. The latter is similar to an ACL-RSI item which asks “are you confident about your ability to perform well at your sport”. Although there are no prior studies directly assessing the correlation between these two constructs, Kim et al. demonstrated an association between ACL-RSI and the KOOS quality of life subscale [34]. Although KOOS and ACL-RSI may not be completely orthogonal instruments, the specific items do capture distinct domains of patient health and further research is warranted to clarify whether favorable patient-perceived knee function indeed may lead to improved psychological readiness. We are unable to quantify the contribution of overlapping item content directly in this analysis, because the item-level scores were not retained. Further studies could explore discriminant validity for patient-perceived function and psychological readiness to further clarify this relationship. Regardless, perceived function and psychological readiness are both modifiable patient factors, representing a target for perioperative rehabilitation and cognitive therapy for selected patients [35,36,37]. Psychological factors that potentially mediate the relationship between perceived function and readiness, including fear of reinjury, self-efficacy, and pain catastrophizing, were not measured in this study. Incorporating these factors could also help determine whether perceived knee function and psychological readiness are distinct constructs.

In this cross-section of patients at 9- and 12-month follow-ups from ACLR, there was no evidence of an association between any SEBT-derived biomechanical endpoint and psychological readiness during functional testing. This study comprehensively evaluated performance through three distinct approaches, including multiplanar lower extremity kinematics, functional test scoring, and an advanced motion quality index, corroborating and extending the findings of prior studies which also did not identify a significant association between ACL-RSI and objective biomechanical endpoints [16,17]. One potential explanation for this incongruity is that performance on certain functional batteries, such as knee strength and single-leg drop jump, may improve asymptotically after one year while psychological readiness is still highly variable and improving [38,39,40]. For example, Gerfroit et al. demonstrated substantial improvements in ACL-RSI at a 5-year follow-up compared to the time of return to sport [40]. In this analysis, there was still substantial variability in psychological readiness (standard deviation 24.6), with 45% of patients scoring above a high readiness threshold of 75. Another explanation for this discordance is that psychological readiness may be truly distinct from objective biomechanical readiness, which strengthens the argument to independently assess both factors as part of a multi-domain return-to-sport protocol. Lastly, the findings from this study should not be generalized beyond the SEBT. Strength, hop performance, landing and deceleration mechanics, and change-of-direction and cutting tasks were not assessed. The absence of an association between SEBT performance and psychological readiness in this cohort should not be interpreted as evidence that objective biomechanical function in general is unrelated to psychological readiness. A multi-task battery would be required to evaluate this broader question.

Despite the finding that there was no evidence of association between psychological readiness and motion quality, as measured by KDI, routine evaluation of motion quality through time series posture analysis should continue to be evaluated in biomechanical and functional test scoring protocols. It has been previously demonstrated that patients are able to achieve similar scores on functional assessments by employing different postural strategies (i.e., different patterns of lower extremity kinematics) [41]. Therefore, patients with persistent impairment may be able to compensate through other joints to achieve the same score. Some motion quality scoring algorithms directly address this by assigning a score via comparison to a control population [42]. Further research is warranted to better understand specifically which compensatory mechanisms tend to be employed during functional assessments in the ACLR population, which could identify potential targets for future return to sport biomechanical biomarkers. By restricting analyses to specific kinematic measures or functional test outputs, it is possible to miss underlying postural differences in different populations.

There are important clinical implications to the finding that patient-perceived knee function was most strongly associated with psychological readiness. Previous studies have established an association between psychological readiness and return to sport success and risk for reinjury [13,14,17]. Although the exact directional relationship between KOOS-12 and ACL-RSI is not known, it is clear there is an association at the time of return-to-sport decision-making. Furthermore, prior studies have established that both ACL-RSI and KOOS-12 are modifiable in the ACLR population, either in response to targeted cognitive behavioral therapy or perioperative physical therapy, and therefore represent a target for intervention in selected patients [35,36,37]. Further research in larger sample sizes is also warranted to investigate patients who have discordant objective and perceived function, as they may represent potential candidates for targeted return-to-sport interventions, including rehabilitative therapy or cognitive therapy. The results of this study are most applicable to patients at the time of return-to-sport decision-making, which is usually 9 to 12 months following ACLR, and strengthen the case for multi-domain return-to-sport criteria. However, there is some evidence that deficits in biomechanical function and psychological readiness are observable as soon as 3–6 months postoperatively [3,43,44]. Further longitudinal studies may add clinical utility by identifying when recovery trajectories begin to diverge and when intervention is first possible. Importantly, analyzing the 9- and 12-month timepoints separately did not change the direction of the primary association, which held at both timepoints (9-month visit, *n* = 23, *r* = 0.68; 12-month visit, *n* = 17, *r* = 0.43), although each visit is individually underpowered. Psychological readiness itself did not differ between the two visit timepoints (*p* = 0.43).

There are several limitations to consider for this study. First, this study did not measure other factors potentially related to reinjury after ACLR including limb alignment, bone morphology, specific sport participation, adherence to rehabilitation, or time from injury to surgery. The sample size was not powered for subanalyses based on graft type, concomitant procedures, and injury timing, which are all factors that may influence recovery timing. Although all patients were documented as having participated in physical therapy in clinical notes, the exact number of sessions attended and home exercise compliance are not known. This cross-sectional study evaluated subjective and SEBT functional performance at the time of return to sport and does not include longitudinal data, which may reveal temporal trends in various domains of ACLR recovery. In this study, KDI had poor within-session reliability compared to the other biomechanical domains. To further evaluate, a sensitivity analysis was performed using the last SEBT trial for each patient (rather than averaging multiple trials) which also did not identify a statistically significant association with psychological readiness (*p* > 0.05). Measurement error may attenuate associations, so the near-null association between motion quality and psychological readiness is potentially partially attributable to unreliable measurement of the predictor rather than the absence of a relationship. Correcting the observed correlation for attenuation, using the observed within-session reliability of the index and the published internal consistency of the ACL-RSI, raises the estimate from *r* = 0.16 to *r* = 0.28. Alternative approaches to motion quality may eventually offer more reproducible measurements.

Although SEBT is commonly used in the return-to-sport setting as it requires high strength, neuromotor coordination, and range of motion, it may be less relevant to specific sports or patient populations, and other functional tests may be more appropriate. Performance on the SEBT and other functional tasks may vary according to specific sport participation, particularly if a sport requires highly asymmetric demands. Furthermore, limb dominance was not recorded, and the nonoperative limbs should not be regarded as “uninjured”, since bilateral neuromuscular deficits exist after ACL injury. Finally, the two selected patient-reported outcome measures, ACL-RSI and KOOS, although employing generally unique items, may not be completely orthogonal instruments and further research may help clarify more specifically the relationship between them. The KOOS-12 was administered in full and scored, but item-level responses were not retained for analysis.

## 5. Conclusions

In this cross-sectional cohort, psychological readiness after ACL reconstruction more closely aligned with patient-perceived knee function than biomechanical performance on the SEBT, indicating that perceived function and biomechanical performance on certain tasks may be divergent at the time of return-to-sport clearance. These findings support the routine inclusion of patient-reported outcome measures alongside functional testing in multi-domain return-to-sport assessment.

## Figures and Tables

**Table 1:** Cohort demographics and surgical information.

Parameter	N (%) or Mean (SD)
Age (years)	32.7 (10.5)
Body Mass Index (kg/m^2^)	25.9 (3.7)
Sex (Female)	22 (55%)
Additional Procedures	
Lateral Extra-articular Tenodesis	6 (15%)
Lateral Meniscus Repair	12 (30%)
Medial Meniscus Repair	4 (10%)
ACL RSI	65.0 (24.6)
High ACL RSI (≥75)	18 (45%)
Low ACL RSI (<75)	22 (55%)
Graft Type	
Allograft	8 (20%)
Autograft	32 (80%)
Hamstring	23
Quadriceps	7
Bone–Tendon–Bone	2
Graft Diameter (mm)	8.8 (0.6)

## Data Availability

Data are unable to be publicly shared due to patient privacy and institutional regulations.

## Supplementary Materials

The following supporting information can be downloaded at: https://www.mdpi.com/article/10.3390/bioengineering13091028/s1, Table S1: Associations between specific SEBT reach directions and psychological readiness. Table S2: Standardised coefficients from the combined multivariable model of ACL-RSI score. Table S3: Pearson correlation matrix for all study variables.
