# Supplementary material for: Patient-Reported Knee Function Versus Star Excursion Balance Performance as Correlates of Psychological Return-to-Sport Readiness After Anterior Cruciate Ligament Reconstruction
Source: Bioengineering (Basel). 2026 Sep 3;13(9):1028. doi: 10.3390/bioengineering13091028 (PMC13603582; doi:10.3390/bioengineering13091028)
Supplement: Supplementary file 1 [file bioengineering-13-01028-s001.zip › bioengineering-4500946-supplementary.pdf]

Supplementary Materials

Supplementary Table S1. Associations between Specific SEBT Reach Directions and Psychological Readiness.

Domain	Direction	ACL-RSI r (95% CI)	ACL-RSI p
Reach	Anterior	0.2 (-0.1, 0.5)	0.18
Reach	Anteromedial	0.1 (-0.2, 0.4)	0.48
Reach	Medial	0.2 (-0.1, 0.5)	0.21
Reach	Posteromedial	0.2 (-0.1, 0.5)	0.22
Reach	Posterior	0.2 (-0.1, 0.5)	0.17
Reach	Posterolateral	0.1 (-0.2, 0.4)	0.56
Reach	Lateral	0.2 (-0.1, 0.5)	0.15
Reach	Anterolateral	0.2 (-0.1, 0.5)	0.29
Knee flexion	Anterior	0.1 (-0.2, 0.4)	0.42
Knee flexion	Anteromedial	0.0 (-0.3, 0.3)	0.91
Knee flexion	Medial	0.1 (-0.3, 0.4)	0.69
Knee flexion	Posteromedial	0.1 (-0.2, 0.4)	0.37
Knee flexion	Posterior	-0.1 (-0.4, 0.3)	0.76
Knee flexion	Posterolateral	0.1 (-0.3, 0.4)	0.7
Knee flexion	Lateral	0.1 (-0.2, 0.4)	0.6
Knee flexion	Anterolateral	-0.1 (-0.4, 0.2)	0.58
Hip flexion	Anterior	0.0 (-0.3, 0.3)	0.89
Hip flexion	Anteromedial	0.0 (-0.3, 0.3)	0.82
Hip flexion	Medial	-0.1 (-0.4, 0.2)	0.57
Hip flexion	Posteromedial	-0.1 (-0.4, 0.2)	0.38
Hip flexion	Posterior	0.0 (-0.3, 0.3)	0.94
Hip flexion	Posterolateral	0.0 (-0.3, 0.3)	0.94
Hip flexion	Lateral	0.0 (-0.3, 0.3)	0.82
Hip flexion	Anterolateral	0.0 (-0.3, 0.3)	0.83
Ankle flexion	Anterior	0.1 (-0.2, 0.4)	0.62
Ankle flexion	Anteromedial	-0.2 (-0.5, 0.1)	0.28
Ankle flexion	Medial	0.1 (-0.2, 0.4)	0.66
Ankle flexion	Posteromedial	-0.1 (-0.4, 0.3)	0.76
Ankle flexion	Posterior	0.1 (-0.2, 0.4)	0.58
Ankle flexion	Posterolateral	0.1 (-0.2, 0.4)	0.62
Ankle flexion	Lateral	0.2 (-0.1, 0.5)	0.22
Ankle flexion	Anterolateral	0.1 (-0.2, 0.4)	0.49
Knee valgus	Anterior	0.3 (0.0, 0.6)	0.05
Knee valgus	Anteromedial	0.1 (-0.2, 0.4)	0.53
Knee valgus	Medial	0.2 (-0.1, 0.5)	0.18
Knee valgus	Posteromedial	0.1 (-0.2, 0.4)	0.45
Knee valgus	Posterior	-0.1 (-0.4, 0.2)	0.6
Knee valgus	Posterolateral	0.0 (-0.3, 0.3)	0.92
Knee valgus	Lateral	0.1 (-0.3, 0.4)	0.72
Knee valgus	Anterolateral	0.1 (-0.2, 0.4)	0.6
KDI	Anterior	0.1 (-0.2, 0.4)	0.61
KDI	Anteromedial	0.1 (-0.3, 0.4)	0.74

KDI	Medial	0.2 (-0.1, 0.5)	0.29
KDI	Posteromedial	0.0 (-0.3, 0.3)	0.82
KDI	Posterior	0.2 (-0.1, 0.5)	0.28
KDI	Posterolateral	0.1 (-0.2, 0.4)	0.66
KDI	Lateral	0.2 (-0.1, 0.5)	0.14
KDI	Anterolateral	-0.1 (-0.4, 0.3)	0.69

Supplementary Table S2. Standardised coefficients from the combined multivariable model of ACL-RSI score. All variables were standardised (mean 0, SD 1) prior to model fitting.

Predictor	*β*	SE	95% CI	*t*	*p*	VIF
Intercept	0.000	0.130	−0.267 to 0.267	0.00	1.000	—
KOOS-12 summary score	0.627	0.146	0.327 to 0.927	4.29	<0.001	1.23
SEBT reach composite	0.215	0.264	−0.328 to 0.757	0.81	0.424	4.02
KDI composite	−0.012	0.167	−0.356 to 0.331	−0.07	0.942	1.61
Knee flexion	0.095	0.237	−0.391 to 0.581	0.40	0.691	3.22
Hip flexion	0.161	0.285	−0.424 to 0.746	0.56	0.578	4.67
Ankle flexion	−0.130	0.151	−0.440 to 0.179	−0.86	0.395	1.31
Knee valgus	0.250	0.329	−0.426 to 0.925	0.76	0.455	6.23
Age	0.058	0.147	−0.243 to 0.359	0.39	0.697	1.24
Sex (female)	0.286	0.190	−0.102 to 0.675	1.51	0.142	2.06
Body mass index	0.075	0.187	−0.309 to 0.459	0.40	0.694	2.01
Leg length	0.194	0.171	−0.157 to 0.546	1.13	0.266	1.69

Table S3. Pearson correlation matrix for all study variables (n = 40). Values are correlation coefficients. Significance is indicated by superscript markers for both uncorrected and multiplicity-adjusted tests.

Variable	1	2	3	4	5	6	7	8	9	10	11	12
1. ACL-RSI	—											
2. KOOS-12	0.57**†	—										
3. SEBT reach composite	0.21	0.09	—									
4. KDI composite	0.16	0.20	0.28	—								
5. Knee flexion	0.06	−0.01	0.03	−0.33*	—							
6. Hip flexion	−0.04	0.10	−0.55**†	0.12	−0.55**†	—						
7. Ankle flexion	0.08	0.24	0.23	0.26	0.06	−0.24	—					
8. Knee valgus	0.18	0.03	0.70**†	0.19	0.50**	−0.66**†	0.27	—				
9. Age	−0.02	−0.09	0.14	−0.09	0.00	−0.10	−0.02	−0.05	—			
10. Sex (female)	0.06	−0.04	−0.41**	−0.05	−0.20	0.14	−0.18	−0.44**	−0.16	—		
11. Body mass index	0.17	0.21	−0.13	0.17	0.15	0.34*	0.02	0.15	−0.07	−0.12	—	
12. Leg length	0.03	0.15	0.14	−0.04	−0.12	−0.06	0.14	−0.13	−0.01	−0.35*	−0.33*	—

ACL-RSI, Anterior Cruciate Ligament Return to Sport after Injury scale; KDI, Kinematic Deviation Index; KOOS-12, Knee injury and Osteoarthritis Outcome Score (12-item); SEBT, star excursion balance test. Kinematic variables (rows 5–8) are operative-limb values recorded during the SEBT. Correlations involving sex are point-biserial (female = 1, male = 0).

* $p < 0.05$ and ** $p < 0.01$, uncorrected. † Holm-adjusted $p < 0.05$ across all 66 pairwise comparisons. Five associations survive adjustment for multiplicity: KOOS-12 with ACL-RSI (adjusted p = 0.007), and four associations among the biomechanical variables themselves (reach composite with knee valgus, adjusted $p < 0.001$; hip flexion with knee valgus, adjusted $p < 0.001$; hip flexion with reach composite, adjusted p = 0.020; hip flexion with knee flexion, adjusted p = 0.021).
